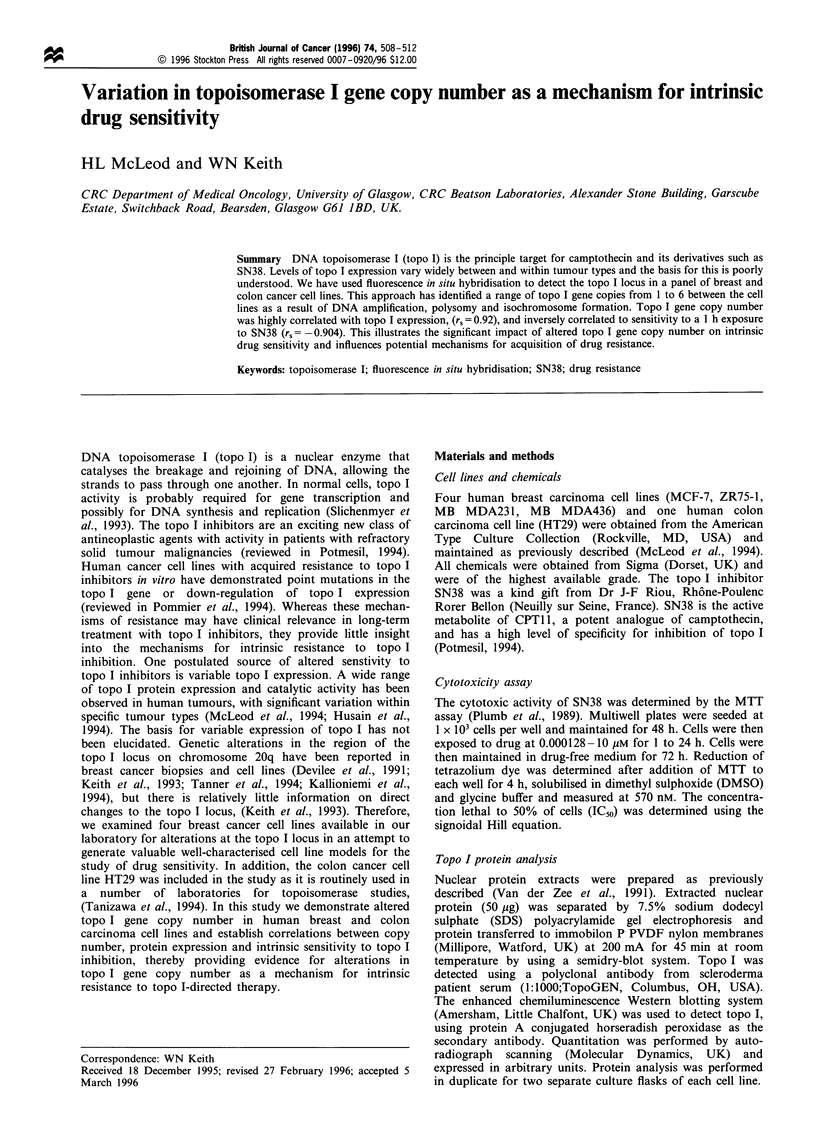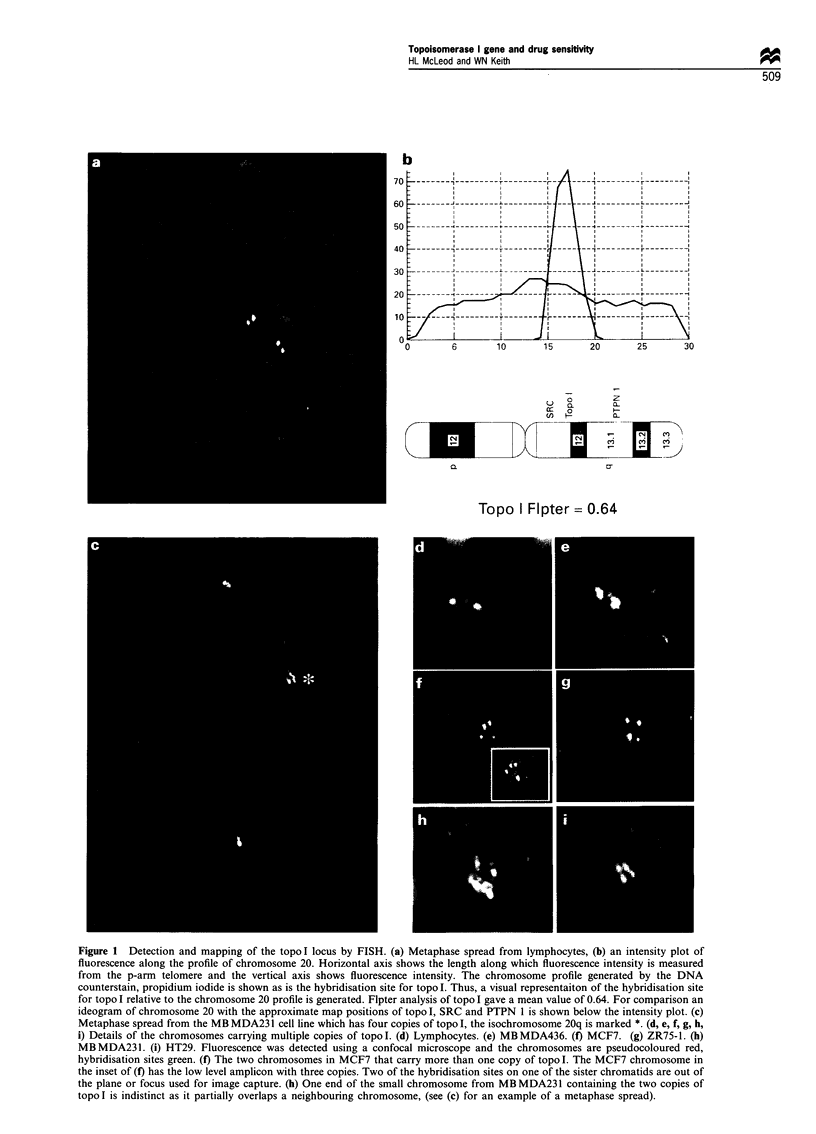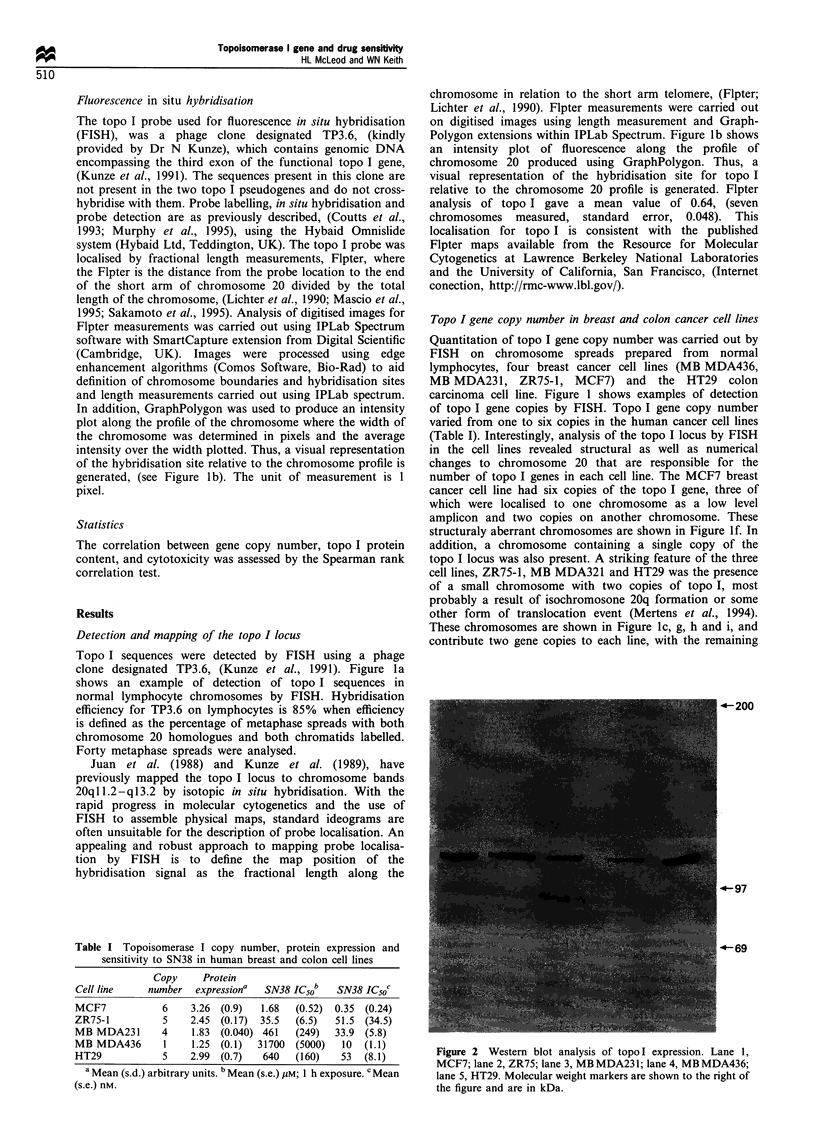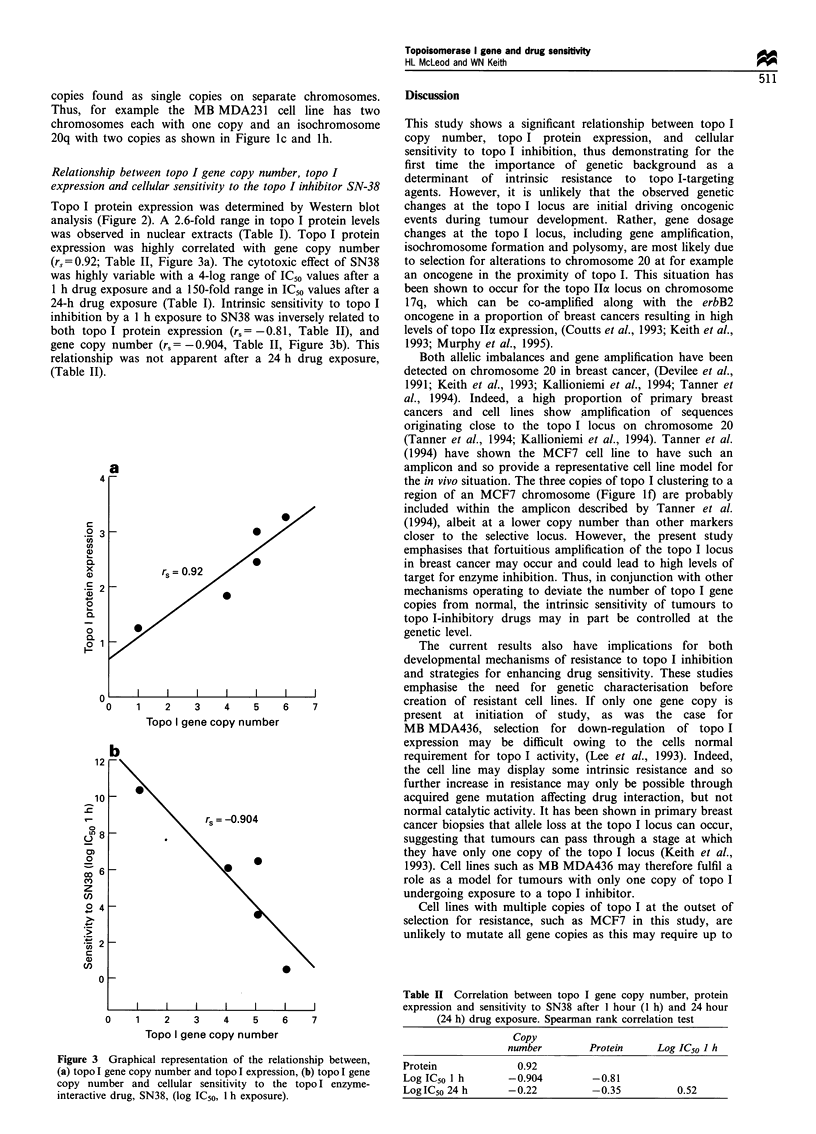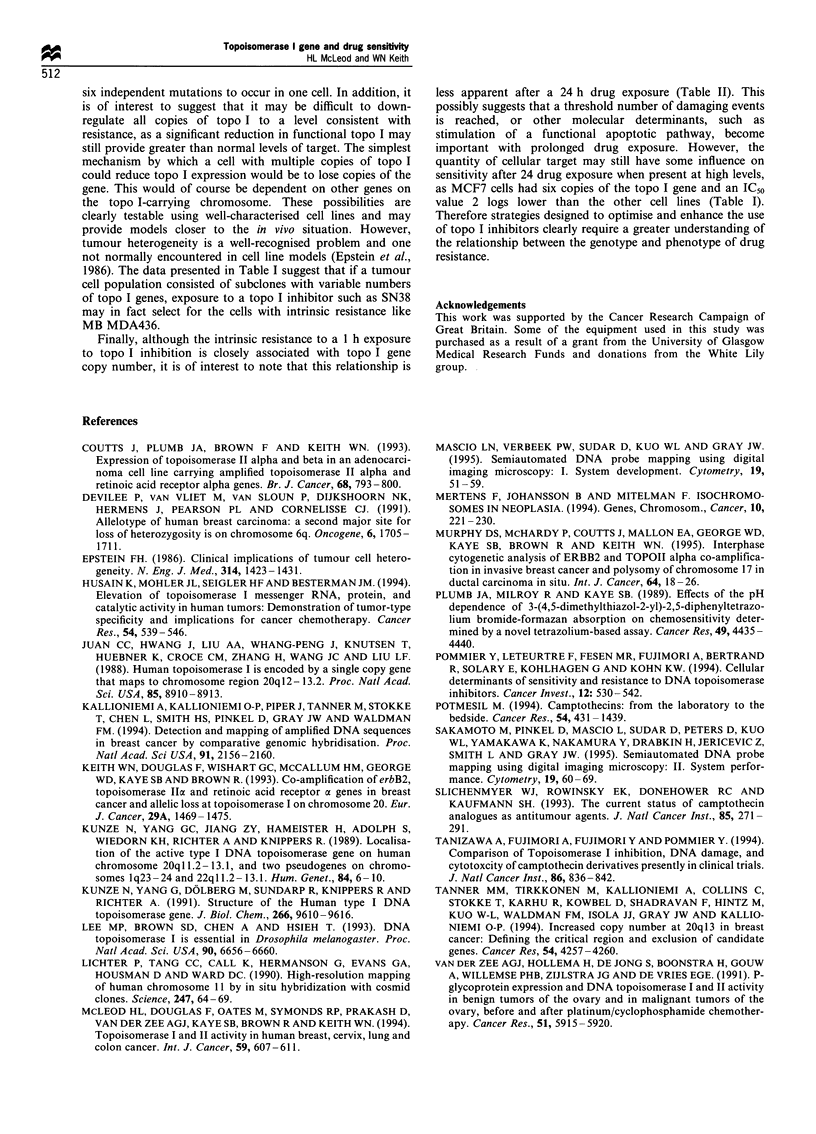# Variation in topoisomerase I gene copy number as a mechanism for intrinsic drug sensitivity.

**DOI:** 10.1038/bjc.1996.394

**Published:** 1996-08

**Authors:** H. L. McLeod, W. N. Keith

**Affiliations:** CRC Department of Medical Oncology, University of Glasgow, UK.

## Abstract

**Images:**


British Journal of Cancer (1996) 74, 508–512
© 1996 Stockton Press All rights reserved 0007–0920/96 $12.00

### Variation in topoisomerase I gene copy number as a mechanism for intrinsic drug sensitivity

HL McLeod and WN Keith

*CRC Department of Medical Oncology, University of Glasgow, CRC Beatson Laboratories, Alexander Stone Building, Garscube Estate, Switchback Road, Bearsden, Glasgow G61 1BD, UK.*

**Summary** DNA topoisomerase I (topo I) is the principle target for camptothecin and its derivatives such as SN38. Levels of topo I expression vary widely between and within tumour types and the basis for this is poorly understood. We have used fluorescence *in situ* hybridisation to detect the topo I locus in a panel of breast and colon cancer cell lines. This approach has identified a range of topo I gene copies from 1 to 6 between the cell lines as a result of DNA amplification, polysomy and isochromosome formation. Topo I gene copy number was highly correlated with topo I expression, ($r_s = 0.92$), and inversely correlated to sensitivity to a 1 h exposure to SN38 ($r_s = -0.904$). This illustrates the significant impact of altered topo I gene copy number on intrinsic drug sensitivity and influences potential mechanisms for acquisition of drug resistance.

**Keywords:** topoisomerase I; fluorescence *in situ* hybridisation; SN38; drug resistance

DNA topoisomerase I (topo I) is a nuclear enzyme that catalyses the breakage and rejoining of DNA, allowing the strands to pass through one another. In normal cells, topo I activity is probably required for gene transcription and possibly for DNA synthesis and replication (Slichenmyer *et al.*, 1993). The topo I inhibitors are an exciting new class of antineoplastic agents with activity in patients with refractory solid tumour malignancies (reviewed in Potmesil, 1994). Human cancer cell lines with acquired resistance to topo I inhibitors *in vitro* have demonstrated point mutations in the topo I gene or down-regulation of topo I expression (reviewed in Pommier *et al.*, 1994). Whereas these mechanisms of resistance may have clinical relevance in long-term treatment with topo I inhibitors, they provide little insight into the mechanisms for intrinsic resistance to topo I inhibition. One postulated source of altered senstivity to topo I inhibitors is variable topo I expression. A wide range of topo I protein expression and catalytic activity has been observed in human tumours, with significant variation within specific tumour types (McLeod *et al.*, 1994; Husain *et al.*, 1994). The basis for variable expression of topo I has not been elucidated. Genetic alterations in the region of the topo I locus on chromosome 20q have been reported in breast cancer biopsies and cell lines (Devilee *et al.*, 1991; Keith *et al.*, 1993; Tanner *et al.*, 1994; Kallioniemi *et al.*, 1994), but there is relatively little information on direct changes to the topo I locus, (Keith *et al.*, 1993). Therefore, we examined four breast cancer cell lines available in our laboratory for alterations at the topo I locus in an attempt to generate valuable well-characterised cell line models for the study of drug sensitivity. In addition, the colon cancer cell line HT29 was included in the study as it is routinely used in a number of laboratories for topoisomerase studies, (Tanizawa *et al.*, 1994). In this study we demonstrate altered topo I gene copy number in human breast and colon carcinoma cell lines and establish correlations between copy number, protein expression and intrinsic sensitivity to topo I inhibition, thereby providing evidence for alterations in topo I gene copy number as a mechanism for intrinsic resistance to topo I-directed therapy.

Correspondence: WN Keith
Received 18 December 1995; revised 27 February 1996; accepted 5 March 1996

### Materials and methods

### Cell lines and chemicals

Four human breast carcinoma cell lines (MCF-7, ZR75-1, MB MDA231, MB MDA436) and one human colon carcinoma cell line (HT29) were obtained from the American Type Culture Collection (Rockville, MD, USA) and maintained as previously described (McLeod *et al.*, 1994). All chemicals were obtained from Sigma (Dorset, UK) and were of the highest available grade. The topo I inhibitor SN38 was a kind gift from Dr J-F Riou, Rhône-Poulenc Rorer Bellon (Neuilly sur Seine, France). SN38 is the active metabolite of CPT11, a potent analogue of camptothecin, and has a high level of specificity for inhibition of topo I (Potmesil, 1994).

### Cytotoxicity assay

The cytotoxic activity of SN38 was determined by the MTT assay (Plumb *et al.*, 1989). Multiwell plates were seeded at $1 \times 10^3$ cells per well and maintained for 48 h. Cells were then exposed to drug at 0.000128–10 $\mu$M for 1 to 24 h. Cells were then maintained in drug-free medium for 72 h. Reduction of tetrazolium dye was determined after addition of MTT to each well for 4 h, solubilised in dimethyl sulphoxide (DMSO) and glycine buffer and measured at 570 nM. The concentration lethal to 50% of cells ($IC_{50}$) was determined using the signoidal Hill equation.

### Topo I protein analysis

Nuclear protein extracts were prepared as previously described (Van der Zee *et al.*, 1991). Extracted nuclear protein (50 $\mu$g) was separated by 7.5% sodium dodecyl sulphate (SDS) polyacrylamide gel electrophoresis and protein transferred to immobilon P PVDF nylon membranes (Millipore, Watford, UK) at 200 mA for 45 min at room temperature by using a semidry-blot system. Topo I was detected using a polyclonal antibody from scleroderma patient serum (1:1000;TopoGEN, Columbus, OH, USA). The enhanced chemiluminescence Western blotting system (Amersham, Little Chalfont, UK) was used to detect topo I, using protein A conjugated horseradish peroxidase as the secondary antibody. Quantitation was performed by autoradiograph scanning (Molecular Dynamics, UK) and expressed in arbitrary units. Protein analysis was performed in duplicate for two separate culture flasks of each cell line.

**Figure 1** Detection and mapping of the topo I locus by FISH. (**a**) Metaphase spread from lymphocytes, (**b**) an intensity plot of fluorescence along the profile of chromosome 20. Horizontal axis shows the length along which fluorescence intensity is measured from the p-arm telomere and the vertical axis shows fluorescence intensity. The chromosome profile generated by the DNA counterstain, propidium iodide is shown as is the hybridisation site for topo I. Thus, a visual representaiton of the hybridisation site for topo I relative to the chromosome 20 profile is generated. Flpter analysis of topo I gave a mean value of 0.64. For comparison an ideogram of chromosome 20 with the approximate map positions of topo I, SRC and PTPN 1 is shown below the intensity plot. (**c**) Metaphase spread from the MB MDA231 cell line which has four copies of topo I, the isochromosome 20q is marked *. (**d, e, f, g, h, i**) Details of the chromosomes carrying multiple copies of topo I. (**d**) Lymphocytes. (**e**) MB MDA436. (**f**) MCF7. (**g**) ZR75-1. (**h**) MB MDA231. (**i**) HT29. Fluorescence was detected using a confocal microscope and the chromosomes are pseudocoloured red, hybridisation sites green. (**f**) The two chromosomes in MCF7 that carry more than one copy of topo I. The MCF7 chromosome in the inset of (**f**) has the low level amplicon with three copies. Two of the hybridisation sites on one of the sister chromatids are out of the plane or focus used for image capture. (**h**) One end of the small chromosome from MB MDA231 containing the two copies of topo I is indistinct as it partially overlaps a neighbouring chromosome, (see (**c**) for an example of a metaphase spread).

*Fluorescence* in situ *hybridisation*

The topo I probe used for fluorescence *in situ* hybridisation (FISH), was a phage clone designated TP3.6, (kindly provided by Dr N Kunze), which contains genomic DNA encompassing the third exon of the functional topo I gene, (Kunze *et al.*, 1991). The sequences present in this clone are not present in the two topo I pseudogenes and do not cross-hybridise with them. Probe labelling, *in situ* hybridisation and probe detection are as previously described, (Coutts *et al.*, 1993; Murphy *et al.*, 1995), using the Hybaid Omnislide system (Hybaid Ltd, Teddington, UK). The topo I probe was localised by fractional length measurements, Flpter, where the Flpter is the distance from the probe location to the end of the short arm of chromosome 20 divided by the total length of the chromosome, (Lichter *et al.*, 1990; Mascio *et al.*, 1995; Sakamoto *et al.*, 1995). Analysis of digitised images for Flpter measurements was carried out using IPLab Spectrum software with SmartCapture extension from Digital Scientific (Cambridge, UK). Images were processed using edge enhancement algorithms (Comos Software, Bio-Rad) to aid definition of chromosome boundaries and hybridisation sites and length measurements carried out using IPLab spectrum. In addition, GraphPolygon was used to produce an intensity plot along the profile of the chromosome where the width of the chromosome was determined in pixels and the average intensity over the width plotted. Thus, a visual representation of the hybridisation site relative to the chromosome profile is generated, (see Figure 1b). The unit of measurement is 1 pixel.

*Statistics*

The correlation between gene copy number, topo I protein content, and cytotoxicity was assessed by the Spearman rank correlation test.

### Results

*Detection and mapping of the topo I locus*

Topo I sequences were detected by FISH using a phage clone designated TP3.6, (Kunze *et al.*, 1991). Figure 1a shows an example of detection of topo I sequences in normal lymphocyte chromosomes by FISH. Hybridisation efficiency for TP3.6 on lymphocytes is 85% when efficiency is defined as the percentage of metaphase spreads with both chromosome 20 homologues and both chromatids labelled. Forty metaphase spreads were analysed.

Juan *et al.* (1988) and Kunze *et al.* (1989), have previously mapped the topo I locus to chromosome bands 20q11.2–q13.2 by isotopic *in situ* hybridisation. With the rapid progress in molecular cytogenetics and the use of FISH to assemble physical maps, standard ideograms are often unsuitable for the description of probe localisation. An appealing and robust approach to mapping probe localisation by FISH is to define the map position of the hybridisation signal as the fractional length along the chromosome in relation to the short arm telomere, (Flpter; Lichter *et al.*, 1990). Flpter measurements were carried out on digitised images using length measurement and GraphPolygon extensions within IPLab Spectrum. Figure 1b shows an intensity plot of fluorescence along the profile of chromosome 20 produced using GraphPolygon. Thus, a visual representation of the hybridisation site for topo I relative to the chromosome 20 profile is generated. Flpter analysis of topo I gave a mean value of 0.64, (seven chromosomes measured, standard error, 0.048). This localisation for topo I is consistent with the published Flpter maps available from the Resource for Molecular Cytogenetics at Lawrence Berkeley National Laboratories and the University of California, San Francisco, (Internet conection, http://rmc-www.lbl.gov/).

*Topo I gene copy number in breast and colon cancer cell lines*

Quantitation of topo I gene copy number was carried out by FISH on chromosome spreads prepared from normal lymphocytes, four breast cancer cell lines (MB MDA436, MB MDA231, ZR75-1, MCF7) and the HT29 colon carcinoma cell line. Figure 1 shows examples of detection of topo I gene copies by FISH. Topo I gene copy number varied from one to six copies in the human cancer cell lines (Table I). Interestingly, analysis of the topo I locus by FISH in the cell lines revealed structural as well as numerical changes to chromosome 20 that are responsible for the number of topo I genes in each cell line. The MCF7 breast cancer cell line had six copies of the topo I gene, three of which were localised to one chromosome as a low level amplicon and two copies on another chromosome. These structuraly aberrant chromosomes are shown in Figure 1f. In addition, a chromosome containing a single copy of the topo I locus was also present. A striking feature of the three cell lines, ZR75-1, MB MDA321 and HT29 was the presence of a small chromosome with two copies of topo I, most probably a result of isochromosone 20q formation or some other form of translocation event (Mertens *et al.*, 1994). These chromosomes are shown in Figure 1c, g, h and i, and contribute two gene copies to each line, with the remaining

**Table I** Topoisomerase I copy number, protein expression and sensitivity to SN38 in human breast and colon cell lines

| *Cell line* | *Copy number* | *Protein expression*[a] | *SN38 $IC_{50}$*[b] | *SN38 $IC_{50}$*[c] |
|---|---|---|---|---|
| MCF7 | 6 | 3.26 (0.9) | 1.68 (0.52) | 0.35 (0.24) |
| ZR75-1 | 5 | 2.45 (0.17) | 35.5 (6.5) | 51.5 (34.5) |
| MB MDA231 | 4 | 1.83 (0.040) | 461 (249) | 33.9 (5.8) |
| MB MDA436 | 1 | 1.25 (0.1) | 31700 (5000) | 10 (1.1) |
| HT29 | 5 | 2.99 (0.7) | 640 (160) | 53 (8.1) |

[a] Mean (s.d.) arbitrary units. [b] Mean (s.e.) μM; 1 h exposure. [c] Mean (s.e.) nM.

**Figure 2** Western blot analysis of topo I expression. Lane 1, MCF7; lane 2, ZR75; lane 3, MB MDA231; lane 4, MB MDA436; lane 5, HT29. Molecular weight markers are shown to the right of the figure and are in kDa.

copies found as single copies on separate chromosomes. Thus, for example the MB MDA231 cell line has two chromosomes each with one copy and an isochromosome 20q with two copies as shown in Figure 1c and 1h.

*Relationship between topo I gene copy number, topo I expression and cellular sensitivity to the topo I inhibitor SN-38*

Topo I protein expression was determined by Western blot analysis (Figure 2). A 2.6-fold range in topo I protein levels was observed in nuclear extracts (Table I). Topo I protein expression was highly correlated with gene copy number ($r_s = 0.92$; Table II, Figure 3a). The cytotoxic effect of SN38 was highly variable with a 4-log range of $IC_{50}$ values after a 1 h drug exposure and a 150-fold range in $IC_{50}$ values after a 24-h drug exposure (Table I). Intrinsic sensitivity to topo I inhibition by a 1 h exposure to SN38 was inversely related to both topo I protein expression ($r_s = -0.81$, Table II), and gene copy number ($r_s = -0.904$, Table II, Figure 3b). This relationship was not apparent after a 24 h drug exposure, (Table II).

**Figure 3** Graphical representation of the relationship between, (**a**) topo I gene copy number and topo I expression, (**b**) topo I gene copy number and cellular sensitivity to the topo I enzyme-interactive drug, SN38, (log $IC_{50}$, 1 h exposure).

**Discussion**

This study shows a significant relationship between topo I copy number, topo I protein expression, and cellular sensitivity to topo I inhibition, thus demonstrating for the first time the importance of genetic background as a determinant of intrinsic resistance to topo I-targeting agents. However, it is unlikely that the observed genetic changes at the topo I locus are initial driving oncogenic events during tumour development. Rather, gene dosage changes at the topo I locus, including gene amplification, isochromosome formation and polysomy, are most likely due to selection for alterations to chromosome 20 at for example an oncogene in the proximity of topo I. This situation has been shown to occur for the topo IIα locus on chromosome 17q, which can be co-amplified along with the *erb*B2 oncogene in a proportion of breast cancers resulting in high levels of topo IIα expression, (Coutts *et al.*, 1993; Keith *et al.*, 1993; Murphy *et al.*, 1995).

Both allelic imbalances and gene amplification have been detected on chromosome 20 in breast cancer, (Devilee *et al.*, 1991; Keith *et al.*, 1993; Kallioniemi *et al.*, 1994; Tanner *et al.*, 1994). Indeed, a high proportion of primary breast cancers and cell lines show amplification of sequences originating close to the topo I locus on chromosome 20 (Tanner *et al.*, 1994; Kallioniemi *et al.*, 1994). Tanner *et al.* (1994) have shown the MCF7 cell line to have such an amplicon and so provide a representative cell line model for the *in vivo* situation. The three copies of topo I clustering to a region of an MCF7 chromosome (Figure 1f) are probably included within the amplicon described by Tanner *et al.* (1994), albeit at a lower copy number than other markers closer to the selective locus. However, the present study emphasises that fortuitious amplification of the topo I locus in breast cancer may occur and could lead to high levels of target for enzyme inhibition. Thus, in conjunction with other mechanisms operating to deviate the number of topo I gene copies from normal, the intrinsic sensitivity of tumours to topo I-inhibitory drugs may in part be controlled at the genetic level.

The current results also have implications for both developmental mechanisms of resistance to topo I inhibition and strategies for enhancing drug sensitivity. These studies emphasise the need for genetic characterisation before creation of resistant cell lines. If only one gene copy is present at initiation of study, as was the case for MB MDA436, selection for down-regulation of topo I expression may be difficult owing to the cells normal requirement for topo I activity, (Lee *et al.*, 1993). Indeed, the cell line may display some intrinsic resistance and so further increase in resistance may only be possible through acquired gene mutation affecting drug interaction, but not normal catalytic activity. It has been shown in primary breast cancer biopsies that allele loss at the topo I locus can occur, suggesting that tumours can pass through a stage at which they have only one copy of the topo I locus (Keith *et al.*, 1993). Cell lines such as MB MDA436 may therefore fulfil a role as a model for tumours with only one copy of topo I undergoing exposure to a topo I inhibitor.

Cell lines with multiple copies of topo I at the outset of selection for resistance, such as MCF7 in this study, are unlikely to mutate all gene copies as this may require up to

**Table II** Correlation between topo I gene copy number, protein expression and sensitivity to SN38 after 1 hour (1 h) and 24 hour (24 h) drug exposure. Spearman rank correlation test

| | *Copy number* | *Protein* | *Log $IC_{50}$ 1 h* |
|---|---|---|---|
| Protein | 0.92 | | |
| Log $IC_{50}$ 1 h | −0.904 | −0.81 | |
| Log $IC_{50}$ 24 h | −0.22 | −0.35 | 0.52 |

six independent mutations to occur in one cell. In addition, it is of interest to suggest that it may be difficult to down-regulate all copies of topo I to a level consistent with resistance, as a significant reduction in functional topo I may still provide greater than normal levels of target. The simplest mechanism by which a cell with multiple copies of topo I could reduce topo I expression would be to lose copies of the gene. This would of course be dependent on other genes on the topo I-carrying chromosome. These possibilities are clearly testable using well-characterised cell lines and may provide models closer to the *in vivo* situation. However, tumour heterogeneity is a well-recognised problem and one not normally encountered in cell line models (Epstein *et al.*, 1986). The data presented in Table I suggest that if a tumour cell population consisted of subclones with variable numbers of topo I genes, exposure to a topo I inhibitor such as SN38 may in fact select for the cells with intrinsic resistance like MB MDA436.

Finally, although the intrinsic resistance to a 1 h exposure to topo I inhibition is closely associated with topo I gene copy number, it is of interest to note that this relationship is less apparent after a 24 h drug exposure (Table II). This possibly suggests that a threshold number of damaging events is reached, or other molecular determinants, such as stimulation of a functional apoptotic pathway, become important with prolonged drug exposure. However, the quantity of cellular target may still have some influence on sensitivity after 24 drug exposure when present at high levels, as MCF7 cells had six copies of the topo I gene and an $IC_{50}$ value 2 logs lower than the other cell lines (Table I). Therefore strategies designed to optimise and enhance the use of topo I inhibitors clearly require a greater understanding of the relationship between the genotype and phenotype of drug resistance.

**Acknowledgements**
This work was supported by the Cancer Research Campaign of Great Britain. Some of the equipment used in this study was purchased as a result of a grant from the University of Glasgow Medical Research Funds and donations from the White Lily group.

### References

COUTTS J, PLUMB JA, BROWN F AND KEITH WN. (1993). Expression of topoisomerase II alpha and beta in an adenocarcinoma cell line carrying amplified topoisomerase II alpha and retinoic acid receptor alpha genes. *Br. J. Cancer*, **68**, 793–800.

DEVILEE P, VAN VLIET M, VAN SLOUN P, DIJKSHOORN NK, HERMENS J, PEARSON PL AND CORNELISSE CJ. (1991). Allelotype of human breast carcinoma: a second major site for loss of heterozygosity is on chromosome 6q. *Oncogene*, **6**, 1705–1711.

EPSTEIN FH. (1986). Clinical implications of tumour cell heterogeneity. *N. Eng. J. Med.*, **314**, 1423–1431.

HUSAIN K, MOHLER JL, SEIGLER HF AND BESTERMAN JM. (1994). Elevation of topoisomerase I messenger RNA, protein, and catalytic activity in human tumors: Demonstration of tumor-type specificity and implications for cancer chemotherapy. *Cancer Res.*, **54**, 539–546.

JUAN CC, HWANG J, LIU AA, WHANG-PENG J, KNUTSEN T, HUEBNER K, CROCE CM, ZHANG H, WANG JC AND LIU LF. (1988). Human topoisomerase I is encoded by a single copy gene that maps to chromosome region 20q12–13.2. *Proc. Natl Acad. Sci. USA*, **85**, 8910–8913.

KALLIONIEMI A, KALLIONIEMI O-P, PIPER J, TANNER M, STOKKE T, CHEN L, SMITH HS, PINKEL D, GRAY JW AND WALDMAN FM. (1994). Detection and mapping of amplified DNA sequences in breast cancer by comparative genomic hybridisation. *Proc. Natl Acad. Sci USA*, **91**, 2156–2160.

KEITH WN, DOUGLAS F, WISHART GC, McCALLUM HM, GEORGE WD, KAYE SB AND BROWN R. (1993). Co-amplification of *erb*B2, topoisomerase IIα and retinoic acid receptor α genes in breast cancer and allelic loss at topoisomerase I on chromosome 20. *Eur. J. Cancer*, **29A**, 1469–1475.

KUNZE N, YANG GC, JIANG ZY, HAMEISTER H, ADOLPH S, WIEDORN KH, RICHTER A AND KNIPPERS R. (1989). Localisation of the active type I DNA topoisomerase gene on human chromosome 20q11.2–13.1, and two pseudogenes on chromosomes 1q23–24 and 22q11.2–13.1. *Hum. Genet.*, **84**, 6–10.

KUNZE N, YANG G, DÖLBERG M, SUNDARP R, KNIPPERS R AND RICHTER A. (1991). Structure of the Human type I DNA topoisomerase gene. *J. Biol. Chem.*, **266**, 9610–9616.

LEE MP, BROWN SD, CHEN A AND HSIEH T. (1993). DNA topoisomerase I is essential in *Drosophila melanogaster*. *Proc. Natl Acad. Sci. USA*, **90**, 6656–6660.

LICHTER P, TANG CC, CALL K, HERMANSON G, EVANS GA, HOUSMAN D AND WARD DC. (1990). High-resolution mapping of human chromosome 11 by in situ hybridization with cosmid clones. *Science*, **247**, 64–69.

McLEOD HL, DOUGLAS F, OATES M, SYMONDS RP, PRAKASH D, VAN DER ZEE AGJ, KAYE SB, BROWN R AND KEITH WN. (1994). Topoisomerase I and II activity in human breast, cervix, lung and colon cancer. *Int. J. Cancer*, **59**, 607–611.

MASCIO LN, VERBEEK PW, SUDAR D, KUO WL AND GRAY JW. (1995). Semiautomated DNA probe mapping using digital imaging microscopy: I. System development. *Cytometry*, **19**, 51–59.

MERTENS F, JOHANSSON B AND MITELMAN F. ISOCHROMOSOMES IN NEOPLASIA. (1994). Genes, Chromosom., *Cancer*, **10**, 221–230.

MURPHY DS, McHARDY P, COUTTS J, MALLON EA, GEORGE WD, KAYE SB, BROWN R AND KEITH WN. (1995). Interphase cytogenetic analysis of ERBB2 and TOPOII alpha co-amplification in invasive breast cancer and polysomy of chromosome 17 in ductal carcinoma in situ. *Int. J. Cancer*, **64**, 18–26.

PLUMB JA, MILROY R AND KAYE SB. (1989). Effects of the pH dependence of 3-(4,5-dimethylthiazol-2-yl)-2,5-diphenyltetrazolium bromide-formazan absorption on chemosensitivity determined by a novel tetrazolium-based assay. *Cancer Res*, **49**, 4435–4440.

POMMIER Y, LETEURTRE F, FESEN MR, FUJIMORI A, BERTRAND R, SOLARY E, KOHLHAGEN G AND KOHN KW. (1994). Cellular determinants of sensitivity and resistance to DNA topoisomerase inhibitors. *Cancer Invest.*, **12**: 530–542.

POTMESIL M. (1994). Camptothecins: from the laboratory to the bedside. *Cancer Res.*, **54**, 431–1439.

SAKAMOTO M, PINKEL D, MASCIO L, SUDAR D, PETERS D, KUO WL, YAMAKAWA K, NAKAMURA Y, DRABKIN H, JERICEVIC Z, SMITH L AND GRAY JW. (1995). Semiautomated DNA probe mapping using digital imaging microscopy: II. System performance. *Cytometry*, **19**, 60–69.

SLICHENMYER WJ, ROWINSKY EK, DONEHOWER RC AND KAUFMANN SH. (1993). The current status of camptothecin analogues as antitumour agents. *J. Natl Cancer Inst.*, **85**, 271–291.

TANIZAWA A, FUJIMORI A, FUJIMORI Y AND POMMIER Y. (1994). Comparison of Topoisomerase I inhibition, DNA damage, and cytotoxcity of camptothecin derivatives presently in clinical trials. *J. Natl Cancer Inst.*, **86**, 836–842.

TANNER MM, TIRKKONEN M, KALLIONIEMI A, COLLINS C, STOKKE T, KARHU R, KOWBEL D, SHADRAVAN F, HINTZ M, KUO W-L, WALDMAN FM, ISOLA JJ, GRAY JW AND KALLIONIEMI O-P. (1994). Increased copy number at 20q13 in breast cancer: Defining the critical region and exclusion of candidate genes. *Cancer Res*, **54**, 4257–4260.

VAN DER ZEE AGJ, HOLLEMA H, DE JONG S, BOONSTRA H, GOUW A, WILLEMSE PHB, ZIJLSTRA JG AND DE VRIES EGE. (1991). P-glycoprotein expression and DNA topoisomerase I and II activity in benign tumors of the ovary and in malignant tumors of the ovary, before and after platinum/cyclophosphamide chemotherapy. *Cancer Res.*, **51**, 5915–5920.